# A randomized double-blind placebo-controlled trial of probiotics in post-surgical colorectal cancer

**DOI:** 10.1186/s12876-019-1047-4

**Published:** 2019-07-24

**Authors:** Liyana Zaharuddin, Norfilza Mohd Mokhtar, Khairul Najmi Muhammad Nawawi, Raja Affendi Raja Ali

**Affiliations:** 1 Department of Physiology, Faculty of Medicine, Universiti Kebangsaan Malaysia, Jalan Yaacob Latif, Bandar Tun Razak, 56000 Cheras, Kuala Lumpur, Malaysia; 2 GUT Research group, Department of Medicine, Faculty of Medicine, Universiti Kebangsaan Malaysia, Jalan Yaacob Latiff, Bandar Tun Razak, 56000 Cheras, Kuala Lumpur, Malaysia

**Keywords:** Colorectal cancer, Probiotic, Cytokines, Lactobacillus spp., Bifidobacterium spp.

## Abstract

**Background:**

Our study aimed to determine the effect of probiotic consumption containing six viable microorganisms of 30 × 10^10^ cfu *Lactobacillus* and *Bifidobacteria* strains for six months on clinical outcomes and inflammatory cytokines (TNF-α, IFN-γ, IL-6, IL-10, IL-12, IL-17A, IL-17C and IL-22) in patients with colorectal cancer.

**Methods:**

Fifty-two patients with colorectal cancer were randomized at four weeks after surgery to receive either a placebo (*n* = 25) or 30 billion colony-forming unit (CFU) of a mixture of six viable strains including 107 mg of *Lactobacillus acidophilus* BCMC® 12,130, *Lactobacillus lactis* BCMC® 12,451, *Lactobacillus casei subsp* BCMC® 12,313, *Bifidobacterium longum* BCMC® 02120, *Bifidobacterium bifidum* BCMC® 02290 and *Bifidobacterium infantis* BCMC® 02129 (*n* = 27). Patients were instructed to take the product orally twice daily for six months. Infection status, diarrhea or hospital admission were recorded throughout the study. Blood was taken pre- and post-intervention to measure TNF-α, IFN-γ, IL-6, IL-10, IL-12, IL-17A, IL-17C and IL-22 using ELISA multiplex kit.

**Results:**

The majority of cases (~ 70%) were in Duke’s C colorectal cancer for both groups. No surgical infection occurred and no antibiotics were required. Chemotherapy induced diarrhea was observed in both groups. Significant reduction in the level of pro-inflammatory cytokine, TNF-α, IL-6, IL-10, IL-12, IL-17A, IL-17C and IL-22 were observed in CRC patients who received probiotics as compared to pre-treatment level (*P* < 0.05). However, there was no significant difference in the IFN-γ in both groups.

**Conclusions:**

We have shown that probiotics containing six viable microorganisms of *Lactobacillus* and *Bifidobacteria* strains are safe to be consumed at four weeks after surgery in colorectal cancer patients and have reduced pro-inflammatory cytokines (except for IFN-gamma). Probiotic may modify intestinal microenvironment resulting in a decline in pro-inflammatory cytokines.

**Trial registration:**

NCT03782428; retrospectively registered on 20th December 2018.

## Background

Colorectal cancer (CRC) is the second most common cancer in males and the third most common in females in most developed countries after discounting non-melanotic skin cancer [1]. It is estimated that colorectal cancer global incidence will reach 2.2 million new cases by the year 2030 [2]. Thus, with this alarming prevalence trend and aggressive nature of the cancer, CRC deserves international attention to further focus on understanding the disease and discover other treatment modalities to curb it.

For decades, functional foods such as probiotic received wide recognition among researchers for its distinct benefits towards gut health [3, 4]. *Lactobacillus* and *Bifidobacteria* were the most commercialized lactic acid probiotics in the market with numbers of published studies supported their direct benefits in preventing CRC [5]. Among clinical benefits of probiotics, pertaining to CRC surgery including reduction in the duration of hospital stay [6], less superficial incisional surgical site infection [7], also preventing chemotherapy and antibiotic induced diarrhoea [8].

The use of probiotic as an adjunct for cancer prevention and treatment was strongly linked with host immune response. In vitro and in vivo studies granted promising evidence on probiotic potential in preventing CRC [9]. For instance, the use of combination of *Lactobacillus acidophilus* ATCC 314 and *Lactobacillus fermentum* NCIMB 5221 reduced cell proliferation and downregulated cellular proliferation marker in the in vivo model of colorectal cancer [9]. Another study reported that *Bifidobacterium adolescentis* SPM0212 significantly impeded cancer growth on three different cell lines Caco-2, SW 480 and HT-29 [10].

Six probiotic strains contained *Streptococcus thermophilus*, *Lactobacillus rhamnosus*, *Lactobacillus acidophilus*, *Lactobacillus casei*, *Bifidobacterium bifidum* and *Bifidobacterium longum* were tested on HT-29 and RKO human colorectal cancer cell lines [11]. All microbial strains when added to the peripheral-blood mononuclear cells co-cultivated with HT-29 cell line caused downregulation of IL-1β, interferon gamma, IL-10 and IL-1 receptor antagonist with no effect on IL-6 and tumor necrosis factor α (TNFα). Similar experiment was done using RKO cell line demonstrated upregulation of TNFα and IL-1β, whereas IL-6, IL-1 receptor antagonist and IL-10 were inhibited at high bacteria to cell ratio [11].

Probiotics ability to modulate the inflammatory factors and enhance immune status inspired researchers to term this specific probiotic genera including the lactic acid probiotics as “immunobiotics” [12]. Unfortunately, studies on “immunobiotics” effects in patients with CRC are very limited. Therefore, this study was aimed to investigate the effect of lactic acid probiotic, *Lactobacillus spp.* and *Bifidobacterium spp.* on the clinical and circulatory cytokines of post-surgical colorectal cancer patients.

## Methods

### Patient recruitment

We performed a randomized, double-blind, placebo-controlled trial involving patients who were 18 years and above, diagnosed with colorectal cancer and planned for colorectal resection in Universiti Kebangsaan Malaysia Medical Centre (UKMMC) from October 2016 to May 2018. We excluded patients, who received antibiotics or consumed pro/pre/synbiotics product two weeks prior to recruitment, patients with recurrent colorectal cancer, advanced metastasis, evidence of recent infections and nursing or pregnant women. The study protocol was approved by the institutional research ethics committee (UKM FPR.4/244/TRGS/2/2014/UKM/02/3) and was registered with the International Clinical Trial Registry Platform (NCT03782428; retrospectively registered on 20th December 2018).

### Clinical assessment of patient

Patients’ clinical characteristics, including age, gender, smoking habits, comorbidities, cancer stage, tumour pathological subtype and site were identified. Patient infection status within six months intervention period was recorded including any reported case of acute gastroenteritis, urinary tract infection, pneumonia, surgical site infection or required any antibiotic administration. Recruited patients were randomized through simple randomization into either treated with probiotic or placebo. Trial unblinding was done upon completion of data analysis. Patients who underwent chemotherapy during the six months intervention period were reviewed and chemotherapy induced diarrhoea were evaluated based on Common Terminology Criteria for Adverse Events version 3.0 (CTCAEv3.0).

### Treatment product and procedure

Probiotics product involved in the study was HEXBIO® manufactured by B-Crobes Laboratories Sdn. Bhd., Malaysia. HEXBIO® contains 30 billion colony-forming unit (CFU) of six viable *Lactobacillus* and *Bifidobacteria* strains, including 107 mg of *Lactobacillus acidophilus* BCMC® 12,130, *Lactobacillus lactis* BCMC® 12,451, *Lactobacillus casei subsp* BCMC® 12,313, *Bifidobacterium longum* BCMC® 02120, *Bifidbacterium bifidum* BCMC® 02290 and *Bifidobacterium infantis* BCMC® 02129. Placebo samples produced were identical to the probiotics in terms of taste and texture except it did not contain any live microorganisms. Both samples were prepared in a form of granules placed in aluminium foil sachets and kept in room temperature. HEXBIO® was labelled as AGE 1 while placebo as AGE 2.

In order to ensure post-surgery antibiotics given will not interfere with the study results, patients were instructed to consume the product four weeks after their surgeries. Patients were required to take the products orally twice daily for six months. We considered good compliance when patients consumed more than 70% of total products given. Anything less than this was regarded as non-compliance.

### Blood samples collection

Prior to surgery, five mL of blood was taken from all the recruited patients. Blood was taken into BD vacutainer and allowed to clot for 30 min. It was then centrifuged for 15 min at 1000Xg where the separated serum was stored in − 80 °C freezer till analyzed. The steps were repeated upon completion of the six months intervention period.

### Enzyme-linked immunosorbent assay (ELISA)

Serum samples were assayed using enzyme-linked immunosorbent assay (ELISA) multiplex kit according to the manufacturer guideline (R&D System Human Magnetic Luminex Assay: LXSAHM-08).

AN amount of 50 μL of Human Magnetic Premixed microparticle cocktail provided in the kit was added to each well of the microplate followed by 50 μL of standards or samples per well. The microplate was then incubated for two hours at room temperature on a horizontal orbital microplate shaker (0.12″ orbit) set at 800 ± 50 rpm. Next, the plate was placed on a designed magnetic device before washed with 100 μL Wash Buffer three times following the manufacturer’s protocol. Later, 50 μL of diluted Biotin Antibody Cocktail was added to each well and incubated for one hour at room temperature on the shaker set at 800 ± 50 rpm followed by the washing step as described earlier. After that, the plate was again re-incubated for 30 min at room temperature on a shaker at 800 ± 50 rpm after 50 μL of diluted Streptavidin-PE was added to each well. The wash step was repeated and finally, 100 μL of Wash Buffer was added in each well before the plate was incubated for two minutes on a shaker at 800 ± 50 rpm. The plate was then read using Luminex® analyser (Luminex Corporation, Austin, TX, USA).

### Statistical analysis

Data were analyzed using SPSS version 22. Quantitative data in the clinicopathological data are expressed as means ± SEM. Measured cytokine level was analyzed using non-parametric Wilcoxon test. P value of less than 0.05 was considered as statistically significant.

## Results

### Patient recruitment

**Fig. 1:**
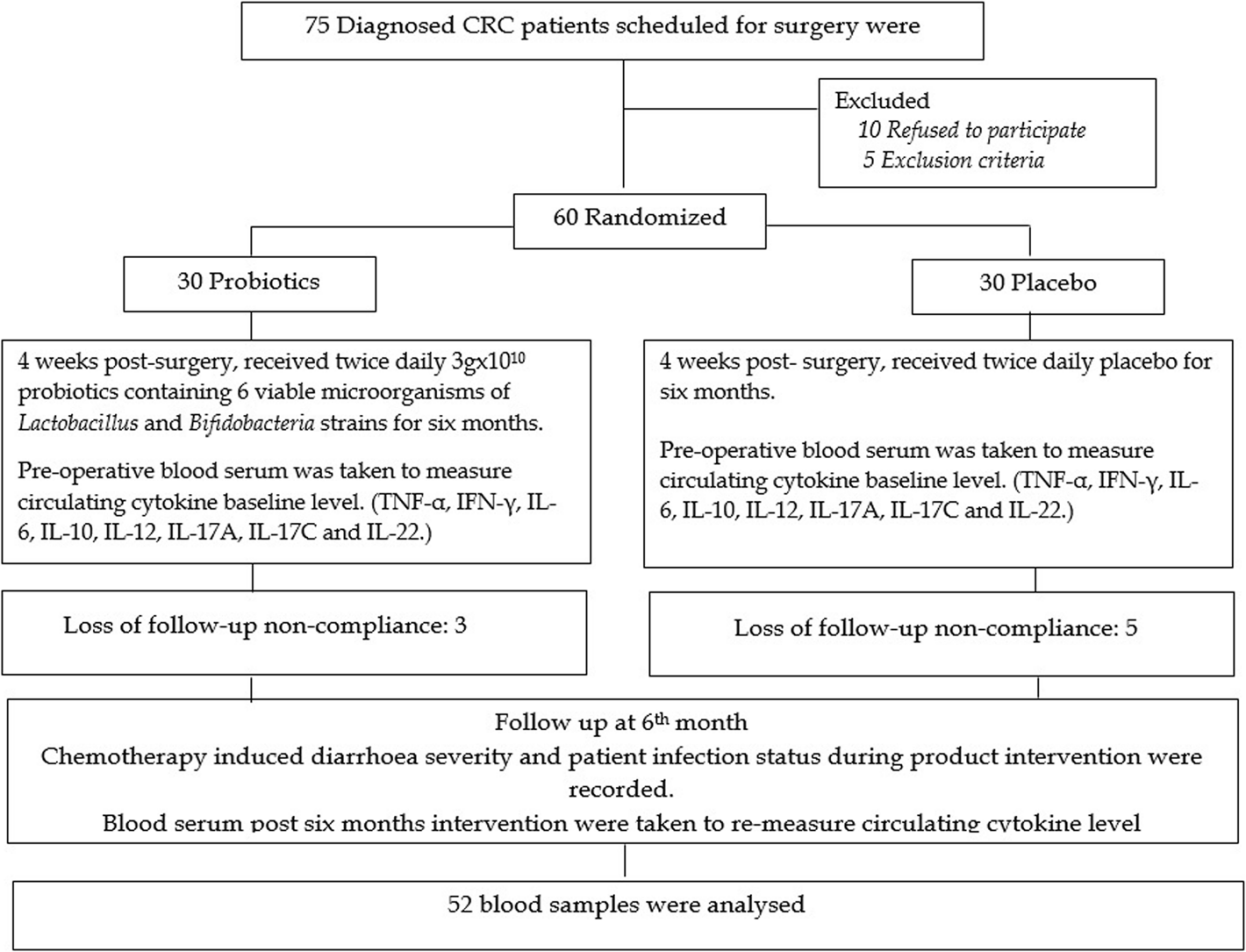
CONSORT diagram of patients’ recruitment and analysis

Seventy five CRC patients scheduled for bowel removal surgery were screened. Only 15 patients were excluded where ten patients refused to participate, and five patients did not meet the inclusion criteria. The remaining 60 patients were equally randomized through simple randomization into either treated with probiotics or placebo. Out of 60 patients recruited, eight patients discontinued the trial due to loss of follow-up or compliance issues. Fifty-two patients completed the trial where twenty-seven patients in the probiotic group and twenty-five patients in the placebo group continued the six months intervention period. The CONSORT diagram of patient recruitment and analysis is shown in Fig. 1.

### Clinical assessment post-intervention of colorectal cancer patients

Table 1 demonstrates the clinicopathological characteristics of recruited patients. Patients’ age, smoking habits and comorbidities was comparable between the two groups. There was no statistically difference in the patients’ ages with the majority was in their 60s. In terms of gender, male was dominated in both groups. The majority of patients were non-smokers. Similarly, there was no significant different in terms of cancer stages, cancer subtype, location and comorbidities between the two groups. Thus, these observations indicated homogeneity among recruited patients.

**Table 1:** Patient clinicopathological characteristics in probiotics and placebo groups

Parameters		Probiotic (*n* = 27)	Placebo (*n* = 25)
Age in years (mean ± SD)		67.33 ± 9.44	66.5 ± 8.57
Gender (n, %)	Male	19 (70.4)	15 (60.0)
	Female	8 (29.6)	10 (40.4)
Smoking status (n, %)	Smoker	8 (29.6)	4 (16.0)
	Ex- smoker	4 (24.8)	10 (56.0)
	Non smoker	15 (55.6)	11 (44.0)
Comorbidities (n, %)	Hypertension	2 (7.4)	2 (8.0)
	Hypertension & Dyslipidaemia	9 (33.3)	9 (36.0)
	Diabetes Mellitus(DM), Hypertension & Dyslipidaemia	2 (7.4)	2 (11.1)
	Chronic Kidney Disease, Hypertension, DM	2 (7.4)	–
	Chronic Heart Disease, Hypertension, Dyslipidaemia	–	1 (4.0)
Stage (n %)	I	1 (.37)	1 (4.0)
	II	8 (29.6)	5 (20.0)
	III	18 (66.7)	19 (76.0)
	IV	–	–
Differentiation	Well differentiated	9 (33.3)	5 (20.0)
	Moderately differentiated	18 (66.7)	20 (80.0)
	Poorly differentiated		
Location	Right	1 (3.7)	1 (4.0)
	Left	26 (96.3)	24 (96.0)
Infection during product consumption (n, %)		–	–
Received adjuvant therapy (n, %)		8 (29.6)	6 (24.0)
Chemotherapy induced diarrhoea (n, %) [CTCAE V 3.0]		6 (22.2)	5 (20.0)

Within six months intervention period, none of the patients reported to have any type of infection or required antibiotic administration. Furthermore, only a small proportion of patients agreed to undergo chemotherapy or adjuvant chemotherapy. Among them, eight patients received probiotics while six patients consumed placebo. Chemotherapy regime involved was XELOX, a combination of Capecitabine and Oxaliplatin. Among these patients, six out of eight patients in the probiotics group and five out of six patients in the placebo group complained of having chemotherapy induced diarrhea. There was no difference in the diarrhea severity scores between the two groups. There was a slight increase in the number of patients who had grade II and grade III diarrhea in both groups (Table 2).

**Table 2:** Chemotherapy induced diarrhoea severity based on Common Terminology Criteria for Adverse Event version 3.0 (CTCAEv3.0) in probiotics and placebo groups

CTCAE Diarrhea Grading	Pre-treatment	Post- treatment
Probiotic (*n* = 6)		
Grade 1 (< 4 times/day)	5	3
Grade 2 (4–6 times/day)	1	2
Grade 3 (> 7 times/day)		1
Grade 4 (life-threatening)		
Grade 5 (death)		
Placebo (*n* = 5)		
Grade 1 (< 4 times/day)	3	1
Grade 2 (4–6 times/day)	2	3
Grade 3 (> 7 times/day)		1
Grade 4 (life-threatening)		
Grade 5 (death)		

### Circulating pro- and anti-inflammatory cytokines in colorectal cancer patients pre- and post-consumption of probiotics

**Fig. 2:**
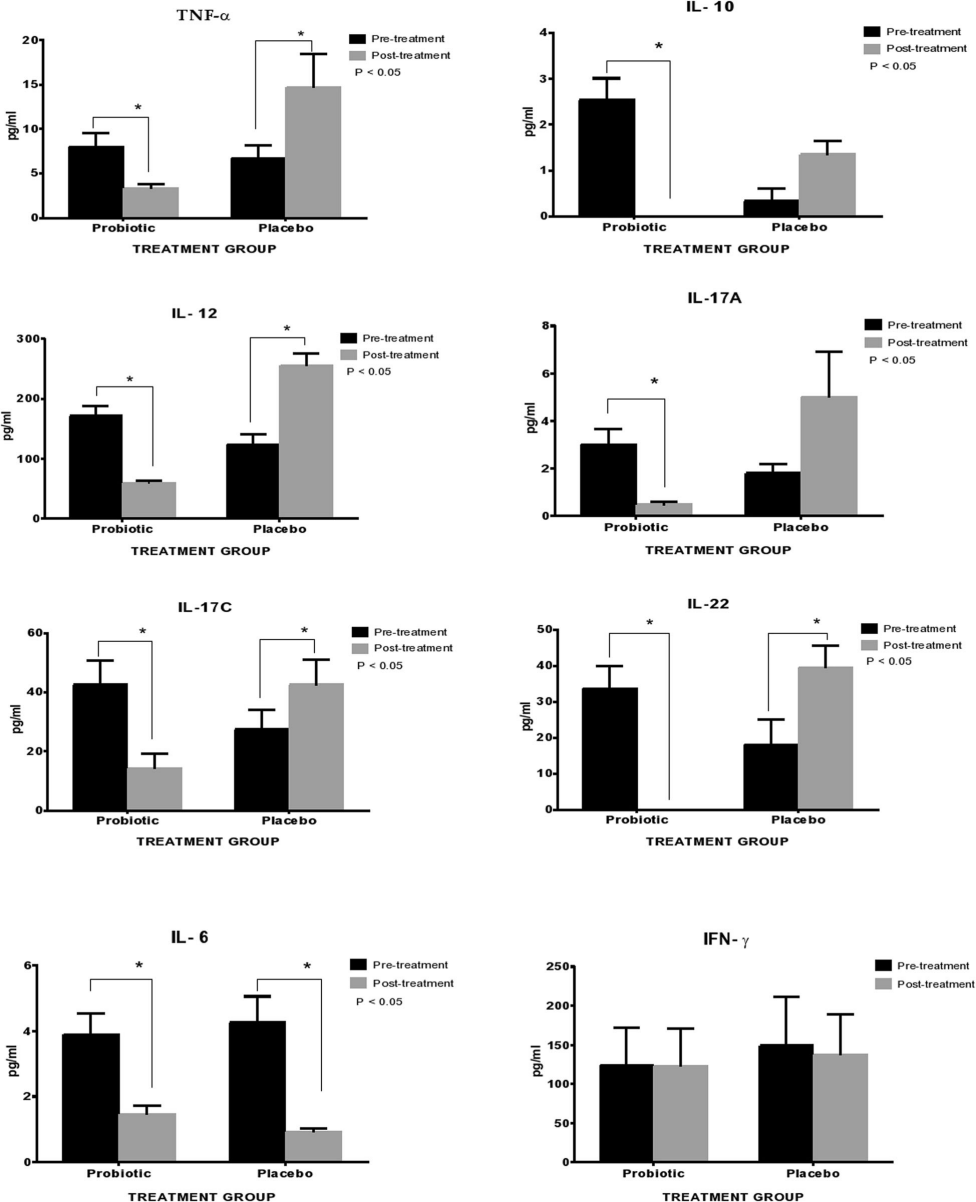
The level of preoperative and post six months inflammatory markers between probiotics and placebo groups. The bar errors indicate the standard error of mean (SEM). Asterik in the figure representing the strength of statistical difference within the groups at *p* < 0.05

Figure 2 demonstrates the level of circulating inflammatory cytokines baseline (pre-operative) and post-intervention following six months of probiotics consumption. The levels of pro-inflammatory cytokines of TNF-α, IL-6, IL-10, IL-12, IL-17A, IL-17C, and IL-22 were significantly reduced among CRC patients as compared to those who received placebo.

In group of patients received probiotic, there was significant lower level of serum TNF-α (*p* = 0.002) post-intervention as compared to the baseline. Similar trends were observed for IL-10 (*p* = 0.028), IL-12 (*p* = 0.005), IL-17A (*p* = 0.00), IL-17C (*p* = 0.018) and IL-22 (*p* = 0.018).

On the other hand, patients who consumed placebo showed significantly increased serum cytokines post intervention as compared to baseline: TNF-α (*p* = 0.005), IL-12 (*p* = 0.028), IL-17C (*p* = 0.028) and IL-22 (*p* = 0.018). The level of IL-10 and IL-17A were slightly increased; however the changes were not statistically significant.

Our finding showed the level of pro-inflammatory IL-6 was significantly reduced in both study groups. After six month post intervention, the level of IL-6 in patients who received probiotics was significantly reduced from 3.88 ± 3.41 pg/mL to 1.44 ± 1.39 pg/mL. Remarkably, the level of IL-6 among patients who received placebo was also significantly reduced from 4.25 ± 4.047 pg/mL to 0.91 ± 0.49 pg/mL. Our findings showed that there was no difference in the level of IFN-γ in pre- and post-intervention of CRC patients that received either probiotic or placebo.

## Discussion

Our study showed that probiotics commenced 4 weeks after surgery for colorectal cancer were safe, did not exacerbate diarrhea, and reduced levels of systemic production of pro-inflammatory cytokines. This is the first local study that provides evidence on lactic acid probiotics are safe for this particular of patients. This finding is supported by a meta-analysis study on the probiotic safety involving 2242 cancer patients [13]. It was estimated that adverse events occurred in 237 patients who consumed probiotic. However, due to varied reporting method it was unclear if the adverse events were link to probiotic related infections [13]. Moreover, the analysis stated that there was no death related to probiotic reported. Therefore, this has concluded that probiotics are safe to be consumed by cancer patients.

In our current clinical trial, there was no difference in the diarrhea severity among patients either taking probiotics or placebo. However, previous study showed that probiotic was also introduced to chemotherapy patients where the trial discovered probiotic possesses the ability to ameliorate chemotherapy induced diarrhea [14]. It was hypothesized that chemotherapy leads to gastrointestinal mucositis through activation of nuclear factor kappa B (NF-κB) pathway thus promoted the released of pro-inflammatory cytokines [14].Various experimental laboratory findings showed probiotic could influence inflammatory cytokines level thus explained it’s protective mechanism on the gastrointestinal layer.

Accumulating evidence showed overexpression of cytokines in serum and tissues were predictive of poor survival among CRC patients and their levels were corresponded to higher risk of CRC [15]. One of the studied cytokines is TNF-α, however the exact biological role of it in cancer promotion is not fully understood. Two possible mechanisms of which it provides resistance to cell death and stimulates production of pro-inflammatory cytokines, including tumor promoting IL-17 cytokine family through the activation of nuclear factor kappa B (NF-κB) and Jun N-terminal kinase (JNK) pathway [16]. Whereas, IL-10 and IL-22 promote tumor cell survival, proliferation and angiogenesis via signal transducer and activator of transcription (STAT) 1,3,5 signalling pathways [17].

Our current study provides evidence that probiotic has the potential to change host inflammatory status thus produces an option for probiotic to be as supplementary for cancer target immunotherapy. In fact, TNF-α and IL-22 antagonist had shown evidence in phase I and phase III clinical trial. However, even with encouraging outcomes, persistent pressing issues raised on the safety of cytokine neutralizing antibody application as a cancer target therapy particularly regarding its concentration and toxicity level [18].

Our current study demonstrated low concentration of IL-10 after consuming six strains of lactic acid probiotics. This finding is in contrast with an in vivo study where administration of probiotic VSL#3 for three weeks caused an early increased in the production of IL-10 as well as high number of regulatory CD4+ T cells bearing surface TGF-beta in the form of latency-associated protein (LAP) in a Th1 T cell murine model of colitis [19]. Furthermore, IL-10 gene deficient mice exhibited normal colonic function with reduced TNF-α production after being treated with VSL#3 for four weeks [20]. It is important to note that although IL-10 is widely acknowledged as anti-inflammatory cytokine, this is only mainly true in laboratory study. In contrast, IL-10 may rather manifest itself as a pro-inflammatory factor in cancer patients.

Similar observation was also seen on IL-12 where IL-12 acts in dual roles as either immune stimulator or immune suppressor. Our clinical study showed the level of IL-12 was not detectable among patients who consumed probiotics six months post-surgery thus supported IL-12 may act as an immune stimulator. This is because IL-12 is a heterodimer cytokine with two unrelated chain composition of p40 and p35 [21]. Furthermore, IL-12p40 is shared to form pro-inflammatory IL-23. High serum concentration of IL-23 was associated with poor outcome and prognosis in patients with colorectal cancer [22].

On the other hand, IL-12 was also found to facilitate the production of anti- inflammatory IFN-γ together with IL-1, IL-2 and IL-15 [23]. In CRC, elevated serum level of IFN-γ was associated with better survival where IFN-γ impeded cell proliferation through phosphorylation of STAT-1 and inhibition of EGFR/Erk1/2 signalling network [24]. Multiple experimental research models demonstrated lactic acid bacteria induced IFN-γ production. For instance, *Lactobacillus acidophilus* was able to increase the level on IFN-γ level in a murine model with induced breast cancer [25]. However, in our trial, consuming probiotics containing *Lactobacillus* and *Bifidobacteria* strains did not influence the level of circulating IFN-γ cytokine. This finding may probably suggest that this particular probiotic cocktail has the potential to influence the level of pro-inflammatory cytokines but limited action towards anti-inflammatory cytokine like IFN-γ.

The reduced level of IL-6 among patients who consumed probiotic in our clinical trial translated similar findings in the recent experimental study on *Lactobacillus fermentum J20, J23* and *J28* in a murine model [26]. Interestingly, we observed similar findings among recruited patients who consumed placebo. This thought provoking finding may reflect multiple roles of IL-6 in human physiology. IL-6 was identified as an inflammatory modulator as well correlated with body muscle-adipose tissue composition, diet, exercise and bone strength [27]. Moreover, another study observed that the level of IL-6 was decreased as soon as 48 h post-surgery compared to their pre-surgery concentration and remained low after five days post-surgery [28]. Therefore, this may suggest that a decreased in IL-6 level post-surgical CRC patients following six months intervention period may be not related to probiotics given but was rather influenced by patients’ physiological status.

Although the exact mechanism of how lactic acid probiotic exerts its anti-inflammatory function is continuously explored, emerging evidence revealed this probiotic has the ability to interfere with the signalling pathways, thus effects, the level of cytokines production [29]. For instance, an in vitro experiment on Lac*tobacillus reuteri* ATCC 6475 reported that it has the potential to downregulate NF-κB dependent gene products which are crucial in cell proliferation and survival [30]. Furthermore, *Lactobacillus acidophilus* 606 was found to cause autophagic cell death on HT-29 cell line through enhancing the Beclin-1 network [31]. While in in vivo study on ob/ob mice treated with probiotic VSL#3 reduced the activity of Jun N-terminal kinase (JNK) [32].

In our clinical trial, circulating inflammatory cytokines of TNF-α, IL-10, IL-12, IL-17A, IL-17C and IL-22 remained high in colorectal cancer patients even after removal of tumor. It was fascinating to note that the level of these cytokines were reduced in recruited patients who consumed probiotics. These findings may further proposed probiotic with high potential to be a promising supplementary for colorectal cancer prevention and treatment. Nevertheless, the application of probiotics in clinical settings remains to be proven in a larger cohort.

## Conclusions

This study provides evidence on lactic acid “immunobiotics” effects in colorectal cancer patients where consumption of probiotics containing 30 billion CFU *Lactobacillus* and *Bifidobacteria* strains twice daily for six months reduced the level of pro-inflammatory cytokines TNF-α, IL-17A, IL-17C, IL-22, IL-10 and IL-12 as well as prevented post-surgical complications in colorectal cancer patients. Furthermore, the trial also proved consumption of probiotics twice daily for six months are safe to be given to CRC patients who underwent surgery and chemotherapy.

## Data Availability

The raw data of the clinical outcomes and ELISA’s results of all cytokines are available from the corresponding author on reasonable request.

## Abbreviations

CRC: Colorectal cancer
ELISA: Enzyme-linked immunosorbent assay
IL: Interleukin
JNK: Jun N-terminal kinase
LAP: Latency-associated protein
NF-κB: Nuclear factor kappa B
STAT: Signal transducer and activator of transcription
TNFα: Tumor necrosis factor α

## References

[1] Ferlay J, Soerjomataram I, Dikshit R, Eser S, Mathers C, Rebelo M, Parkin DM, Forman D, Bray F (2015). Cancer incidence and mortality worldwide: sources, methods and major patterns in GLOBOCAN 2012. Int J Cancer.

[2] Arnold M, Sierra MS, Laversanne M, Soerjomataram I, Jemal A, Bray F (2017). Global patterns and trends in colorectal cancer incidence and mortality. Gut.

[3] Quigley EM (2012). Prebiotics and probiotics: their role in the management of gastrointestinal disorders in adults. Nutr Clin Pract.

[4] Gwee KA, Lee WW, Ling KL, Ooi CJ, Quak SH, Dan YY, Siah KT, Huang JG, Chua ASB, Hilmi IN (2018). Consensus and contentious statements on the use of probiotics in clinical practice: a south east Asian gastro-neuro motility association working team report. J Gastroenterol Hepatol.

[5] Zhong L, Zhang X, Covasa M (2014). Emerging roles of lactic acid bacteria in protection against colorectal cancer. World J Gastroenterol.

[6] Tan CK, Said S, Rajandram R, Wang Z, Roslani AC, Chin KF (2016). Pre-surgical Administration of Microbial Cell Preparation in colorectal Cancer patients: a randomized controlled trial. World J Surg.

[7] Aisu N, Tanimura S, Yamashita Y, Yamashita K, Maki K, Yoshida Y, Sasaki T, Takeno S, Hoshino S (2015). Impact of perioperative probiotic treatment for surgical site infections in patients with colorectal cancer. Exp Ther Med.

[8] Dietrich CG, Kottmann T, Alavi M (2014). Commercially available probiotic drinks containing lactobacillus casei DN-114001 reduce antibiotic-associated diarrhea. World J Gastroenterol.

[9] Kahouli I, Malhotra M, Westfall S, Alaoui-Jamali MA, Prakash S (2017). Design and validation of an orally administrated active L. fermentum-L. acidophilus probiotic formulation using colorectal cancer Apc (min/+) mouse model. Appl Microbiol Biotechnol.

[10] Kim Y, Lee D, Kim D, Cho J, Yang J, Chung M, Kim K, Ha N (2008). Inhibition of proliferation in colon cancer cell lines and harmful enzyme activity of colon bacteria by Bifidobacterium adolescentis SPM0212. Arch Pharm Res.

[11] Djaldetti M, Bessler H (2017). Probiotic strains modulate cytokine production and the immune interplay between human peripheral blood mononucear cells and colon cancer cells. FEMS Microbiol Lett.

[12] Clancy R (2003). Immunobiotics and the probiotic evolution. FEMS Immunol Med Microbiol.

[13] Hassan H, Rompola M, Glaser AW, Kinsey SE, Phillips RS (2018). Systematic review and meta-analysis investigating the efficacy and safety of probiotics in people with cancer. Support Care Cancer.

[14] Touchefeu Y, Montassier E, Nieman K, Gastinne T, Potel G, Bruley d, Varannes S, Le Vacon F, de La Cochetiere MF (2014). Systematic review: the role of the gut microbiota in chemotherapy- or radiation-induced gastrointestinal mucositis - current evidence and potential clinical applications. Aliment Pharmacol Ther.

[15] West NR, McCuaig S, Franchini F, Powrie F (2015). Emerging cytokine networks in colorectal cancer. Nat Rev Immunol.

[16] Balkwill F (2006). TNF-alpha in promotion and progression of cancer. Cancer Metastasis Rev.

[17] Mager LF, Wasmer MH, Rau TT, Krebs P (2016). Cytokine-induced modulation of colorectal Cancer. Front Oncol.

[18] Vacchelli E, Aranda F, Bloy N, Buque A, Cremer I, Eggermont A, Fridman WH, Fucikova J, Galon J, Spisek R (2016). Trial watch-Immunostimulation with cytokines in cancer therapy. Oncoimmunology.

[19] Di Giacinto C, Marinaro M, Sanchez M, Strober W, Boirivant M (2005). Probiotics ameliorate recurrent Th1-mediated murine colitis by inducing IL-10 and IL-10-dependent TGF-beta-bearing regulatory cells. J Immunol.

[20] Madsen K, Cornish A, Soper P, McKaigney C, Jijon H, Yachimec C, Doyle J, Jewell L, De Simone C (2001). Probiotic bacteria enhance murine and human intestinal epithelial barrier function. Gastroenterology.

[21] Trinchieri G (1998). Interleukin-12: a cytokine at the interface of inflammation and immunity. Adv Immunol.

[22] Hu WH, Chen HH, Yen SL, Huang HY, Hsiao CC, Chuang JH (2017). Increased expression of interleukin-23 associated with progression of colorectal cancer. J Surg Oncol.

[23] Chan SH, Perussia B, Gupta JW, Kobayashi M, Pospisil M, Young HA, Wolf SF, Young D, Clark SC, Trinchieri G (1991). Induction of interferon gamma production by natural killer cell stimulatory factor: characterization of the responder cells and synergy with other inducers. J Exp Med.

[24] Wang L, Wang Y, Song Z, Chu J, Qu X (2015). Deficiency of interferon-gamma or its receptor promotes colorectal cancer development. J Interf Cytokine Res.

[25] Maroof H, Hassan ZM, Mobarez AM, Mohamadabadi MA (2012). Lactobacillus acidophilus could modulate the immune response against breast cancer in murine model. J Clin Immunol.

[26] Reyes-Díaz Aline, Mata-Haro Verónica, Hernández Jesús, González-Córdova Aarón, Hernández-Mendoza Adrián, Reyes-Díaz Ricardo, Torres-Llanez María, Beltrán-Barrientos Lilia, Vallejo-Cordoba Belinda (2018). Milk Fermented by Specific Lactobacillus Strains Regulates the Serum Levels of IL-6, TNF-α and IL-10 Cytokines in a LPS-Stimulated Murine Model. Nutrients.

[27] Maggio M, Guralnik JM, Longo DL, Ferrucci L (2006). Interleukin-6 in aging and chronic disease: a magnificent pathway. J Gerontol A Biol Sci Med Sci.

[28] Rettig TC, Verwijmeren L, Dijkstra IM, Boerma D, van de Garde EM, Noordzij PG (2016). Postoperative Interleukin-6 level and early detection of complications after elective major abdominal surgery. Ann Surg.

[29] Thomas CM, Versalovic J (2010). Probiotics-host communication: modulation of signaling pathways in the intestine. Gut Microbes.

[30] Iyer C, Kosters A, Sethi G, Kunnumakkara AB, Aggarwal BB, Versalovic J (2008). Probiotic lactobacillus reuteri promotes TNF-induced apoptosis in human myeloid leukemia-derived cells by modulation of NF-kappaB and MAPK signalling. Cell Microbiol.

[31] Kim Y, Oh S, Yun HS, Oh S, Kim SH (2010). Cell-bound exopolysaccharide from probiotic bacteria induces autophagic cell death of tumour cells. Lett Appl Microbiol.

[32] Li Z, Yang S, Lin H, Huang J, Watkins PA, Moser AB, Desimone C, Song XY, Diehl AM (2003). Probiotics and antibodies to TNF inhibit inflammatory activity and improve nonalcoholic fatty liver disease. Hepatology.

